# The role of matrix stiffness in breast cancer progression: a review

**DOI:** 10.3389/fonc.2023.1284926

**Published:** 2023-10-17

**Authors:** Ruoxi Xu, Peng Yin, Jifu Wei, Qiang Ding

**Affiliations:** ^1^ Department of Pharmacy, The Affiliated Cancer Hospital of Nanjing Medical University, Jiangsu Cancer Hospital, Jiangsu Institute of Cancer Research, Nanjing, China; ^2^ Jiangsu Breast Disease Center, The First Affiliated Hospital with Nanjing Medical University, Nanjing, China

**Keywords:** matrix stiffness, mechanical stimulation, extracellular matrix, breast cancer, signaling pathways

## Abstract

The significance of matrix stiffness in cancer development has been investigated in recent years. The gradual elastic force the extracellular matrix imparts to cells, known as matrix stiffness, is one of the most important types of mechanical stimulation. Increased matrix stiffness alters the biological activity of cells, which promotes the growth of numerous malignancies, including breast cancer. Comprehensive studies have demonstrated that increasing matrix stiffness activates molecular signaling pathways that are closely linked to breast cancer progression. There are many articles exploring the relationship between mechanism hardness and breast cancer, so we wanted to provide a systematic summary of recent research advances. In this review, we briefly introduce the mechanism of matrix stiffness in breast cancer, elaborate on the effect of extracellular matrix stiffness on breast cancer biological behavior and signaling pathways, and finally, we will talk about breast cancer treatment that focuses on matrix stiffness.

## 1 Introduction

Breast cancer is one of the most common malignancies among women. One of the most obvious signs of breast cancer is tissue hardening, which can be detected by palpating malignant nodules (1, 2). The stiffness of normal healthy breast tissue is approximately 0.2 kPa, while that of breast cancer tissue is over 4 kPa (3). In addition to the increased stiffness of breast cancer tissues, adjacent matrix tissues are also affected. A study in an animal model demonstrated that when breast tissue become invasive, the adjacent matrix tissue is also much stiffer than the distant normal tissue (1). These phenomena arise from abnormal changes in the structure and composition of the tumor extracellular matrix (ECM) in breast cancer (2).

The ECM is an intricate network of three-dimensional macromolecules consisting mainly of collagen, non-collagen, elastin, proteoglycans and glycosaminoglycans (4); it offers appropriate chemical signals and mechanical stimulation to regulate cell shape, metabolism, function, migration, proliferation, and differentiation (4, 5). Mechanical stimulation involves compression, matrix stiffness, and hydrodynamics (6). Moreover, matrix stiffness, also known as rigidity or modulus of elasticity, is defined as the resistance of a material to deformation by a force applied at a very slow rate (quasi-static) (7). Stiffness is an intrinsic material property of tissues, and increased tissue hardness is the most obvious and recognized mechanical abnormality in tumors, including breast cancer (8).

With the application of atomic force microscopy (AFM) and the continuous refinement of 3D culture techniques, the study of matrix stiffness has become more feasible (9, 10). Hydrogels are good candidates in the study of ECM physical properties using 3D modeling (11). Various types of hydrogels have been used in the study of matrix stiffness, including polyacrylamide hydrogels, hyaluronic acid hydrogels, collagen hydrogels, gelatin hydrogels, etc. (12). Based on the fact that the matrix stiffness is a constant state of transformation with the dynamics of the ECM, more advanced stimuli-responsive hydrogels were synthesized (13). Stimuli-responsive hydrogels can adjust their stiffness in response to external physical or chemical stimuli to better mimic the *in vivo* environment in matrix stiffness studies (11, 13). In addition, to better approach the treatment of breast cancer from the aspect of stromal stiffness, various 3D experimental models have been developed, mainly including cancer cell lines, 3D spheroids, *in vivo* patient-derived xenografts (PDX), and *in vitro* patient-derived organoids (PDO) (14–17). For example, PDOs have been established from breast cancer, and this model can be used to predict drug response in cancer patients, which in turn informs the patient’s treatment regimen (17). 3D spheroids also have extensive use in exploring the role of matrix stiffness in breast cancer invasion (18).

The formation of tumors mainly depends on the balance between increased matrix stiffness and matrix degradation (19). Collagen accumulation and pathological collagen cross-linking are the major causes of increased ECM stiffness in breast cancer (7). Analysis of human breast tissue samples has revealed that the transition from non-malignant tissue to invasive ductal carcinoma (IDC) corresponds to significant collagen deposition, resulting in stromal stiffening (20). In addition, computational analysis of mammographic images has shown that dense breast tissue has a stiffer matrix, contains more linearized and bound collagen, and is associated with a higher risk of breast cancer (21, 22). Degradation of breast cancer matrix is mainly dependent on the regulation of lysyl oxidase (LOX), lysyl oxidase like-1-4 (LOXL 1-4), and matrix metalloproteinases (MMPs), which are extracellular matrix remodeling enzymes (23, 24). LOX promotes the cross-linking of elastin and collagen in the ECM and prevents collagen degradation, which promotes breast cancer progression (25). Furthermore, MMPs remodel the ECM by degrading ECM proteins, which in turn promote breast cancer metastasis (26). Thus, an excessively stiff matrix or excessive matrix degradation can promote the progression of breast cancer.

Matrix stiffness is closely related to malignant breast cancer phenotypes, including proliferation, metastasis, invasion, and drug resistance. There are many articles exploring the relationship between matrix stiffness and breast cancer, so we want to provide a systematic summary of recent research advances. In this review, we systematically introduce the major causes of breast stiffening and summarize the role of matrix stiffness in breast cancer initiation and progression and its potential applications. This may provide clues for studying matrix stiffness in breast cancer and exploring its clinical applications in breast cancer treatment.

## 2 Formation of matrix stiffness in breast cancer

**Figure 1:**
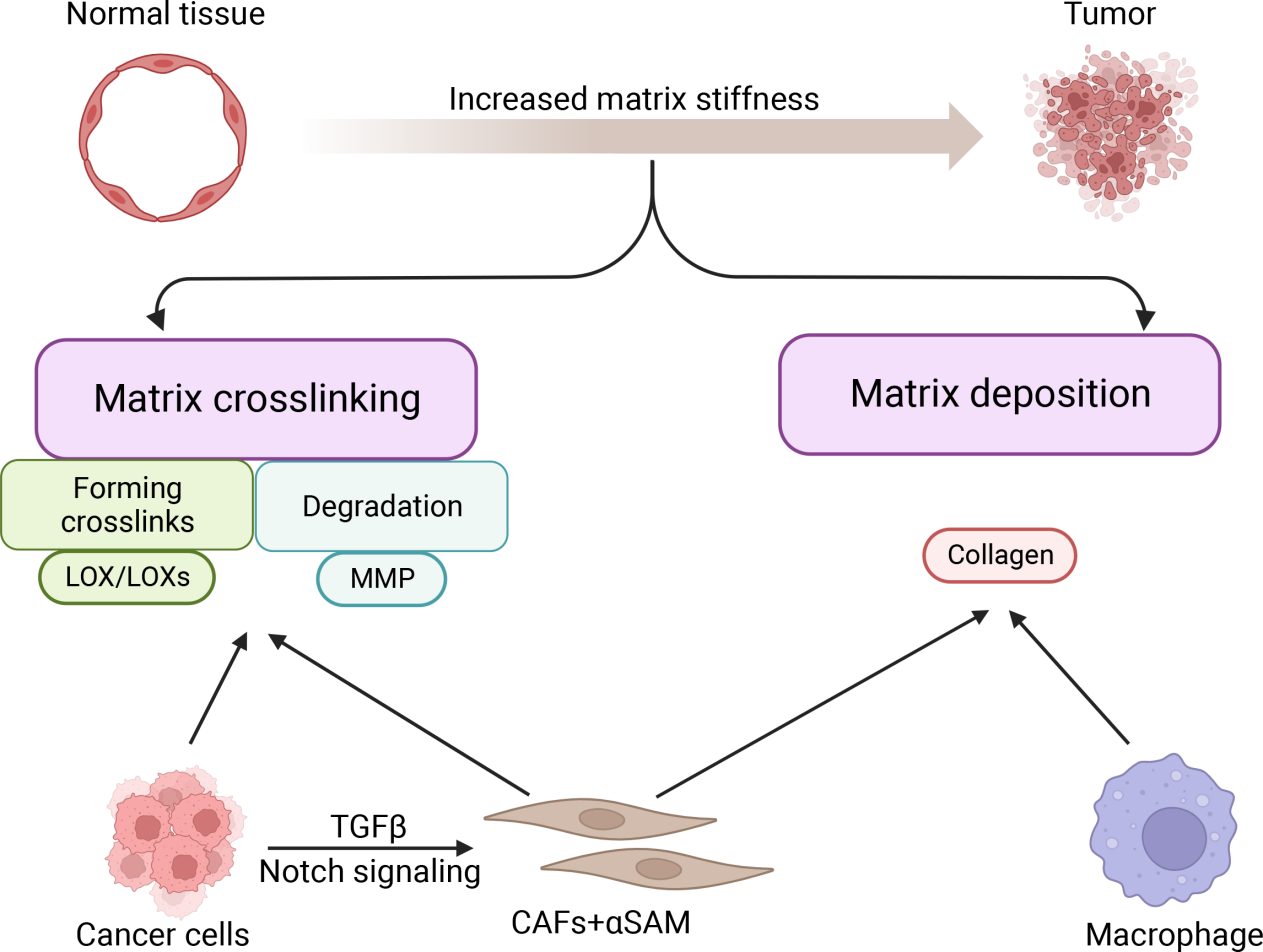
Formation of matrix stiffness in breast cancer.

The stiffness of cancer tissue is mainly determined by cancer and stromal cells (27). Matrix deposition and cross-linking are the two major causes of breast cancer stiffening (28, 29) (**Figure 1**). Cancer cells and stromal cells are jointly involved in matrix deposition and cross-linking and determine matrix stiffness (27).

### 2.1 Matrix deposition

Among all stromal cells, cancer-associated fibroblasts (CAFs) are the most efficient in depositing and remodeling the ECM in the tumor microenvironment (30, 31). Stromal cells with high alpha-smooth muscle actin (aSMA) expression are known as cancer-associated fibroblasts (CAFs) (32). Through Notch signaling, interaction between cancer cells and fibroblasts can advance the CAF phenotype in breast cancer (30). Cancer cells can also promote the transformation of fibroblasts into CAFs by secreting TGFβ, which in turn further promotes tumor progression through ECM remodeling (33, 34). During breast cancer progression, up to 80% of stromal cells acquire the CAF phenotype (35). CAFs synthesize and secrete collagen procollagen molecules, which are processed and arranged to form collagen fibers. As fibrillar collagen (both type I and type III) is progressively deposited in the ECM, the normal ECM gradually transforms into dense fibrous tumor stroma (36, 37). Except for CAFs, other stromal cells play an important role in causing increased matrix stiffness, including macrophages. Macrophages secrete a variety of soluble factors that induce ECM deposition, thereby stiffening the extracellular matrix (20). In addition, during breast cancer progression, breast cancer epithelial cells gradually lose epithelial markers to acquire mesenchymal markers and mesenchymal cell-like properties through epithelial-mesenchymal transition (EMT), which in turn exerts a function like that of CAFs, synthesizing and secreting collagen, leading to stromal deposition promoting an increase in stromal stiffness (38). In summary, both stromal cells and breast cancer cells undergoing EMT can promote increased matrix stiffness through matrix deposition.

### 2.2 Matrix cross-linking

CAFs and cancer cells highly express LOX/LOXs (39), which are amine oxidases that mainly regulate covalent cross-linking between ECM collagen and elastin (40, 41). In breast cancer, LOX and collagen influence the architecture of the ECM and create a favorable microenvironment for tumor development and progression (42).

LOX was reported to promote fibrosis of breast tissue through collagen cross-linking, leading to increased matrix stiffness in breast tumors (43, 44). Increased matrix stiffness can induce the assembly of focal adhesions and up-regulate GFR-dependent PI3K signaling, ultimately leading to tumor progression (1). Furthermore, breast cancer cells and CAFs can synthesize and secrete proteolytically active MMPs (45), which can degrade almost all proteins in the ECM when metal ions are used as cofactors (46). However, when collagen is cross-linked, MMPs are unable to break down the collagen, thus increasing matrix stiffness (47). The phenomenon of collagen cross-linking leading to increased matrix stiffness in cancer cells is widespread. For instance, when highly expressed in pancreatic cancer cells, tissue transglutaminase (TG2) crosslinks proteins to stiffen the pancreatic tumor tissue (48). However, TG2 promotion of breast tumor matrix cross-linking has not been elucidated. In conclusion, matrix cross-linking is essential for enhancing the stiffness of cancer tissues (29).

## 3 Initiation and progression of breast cancer regulated by matrix stiffness

**Figure 2:**
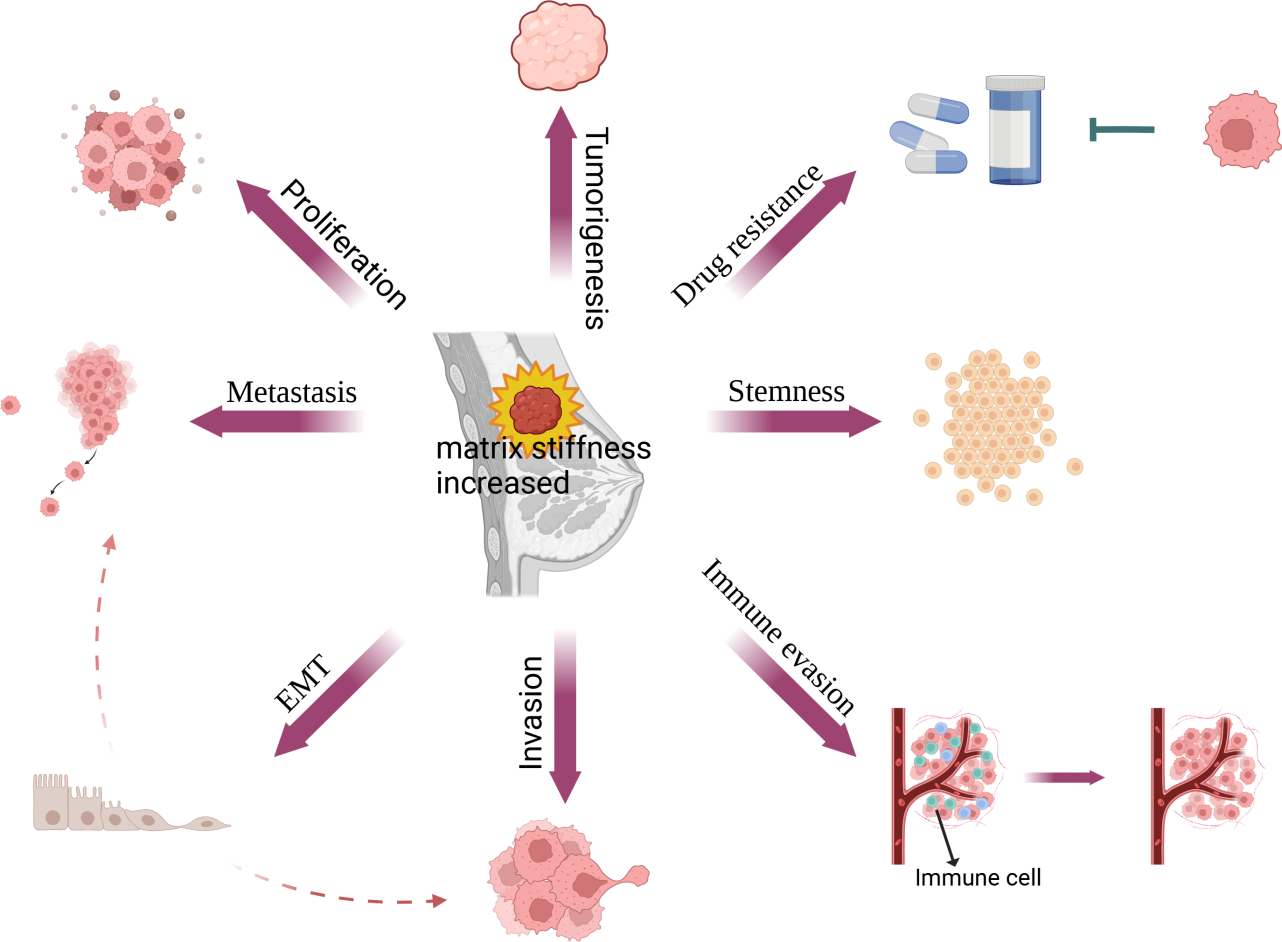
Relationship between matrix stiffness and breast cancer. In breast cancer, matrix stiffness can affect its proliferation, EMT, metastasis, invasion, immune evasion, stemness and drug resistance, thus further promoting fibrosis and the progression of breast cancer.

Increased matrix stiffness leads to breast malignancy and contributes to the malignant phenotypes of breast cancer by promoting breast cancer proliferation, metastasis, invasion, immune evasion, stemness, and drug resistance through the regulation of breast cancer and stromal cells (**Figure 2**). With the development of 3d culture technology, it has become possible to simulate different matrix stiffnesses using hydrogels, making it possible to study *in vitro* how matrix stiffness affects cell signaling pathways. Matrix stiffness affects tumor and non-tumor cells through multiple molecular signaling pathways in breast tumors that promote tumor progression (**Table 1**).

**Table 1:** List of Matrix Stiffness Affecting Breast Cancer.

Phenotype	Signaling Pathway	Effect on Cells	References
Tumorigenesis	ROCK signaling pathway	Rho GTPases activation	(49)
	Integrin signaling pathway	Integrin/PI3K activation, oncogene initiation	(1)
Proliferation	YAP signaling pathway	YAP/MLC upregulation, PASP secretion	(50)
	Wnt signaling pathway	β-catenin upregulation	(51, 52)
	Integrin signaling pathway	ILK-mediated Frizzled-1 upregulation	(53)
	FAK signaling pathway	FAK-Rho upregulation, Ras-MAPK activation	(54)
Invasion/Metastasis	Integrin signaling pathway	Integrin/IR/PI3K/AKT/mTORC1 activation	(55)
		EGFR/PLCγ1 activation, Mena upregulation	(56, 57)
	ROCK signaling pathway	RhoA/ROCK1/p-MLC and RhoA/ROCK2/p-cofilin in a coordinate fashion to modulate breast cancer cell motility	(58)
	TWIST1 signaling pathway	Promoted EMT, TWIST1 nuclear transportation	(59, 60)
Stemness	Integrin signaling pathway	Integrin/ILK/PI3K/Akt activation	(61)
	**\**	TAZ/NANOG dissociate, SOX2 and OCT4 upregulation	(62)
	YAP signaling pathway	YAP nuclear translocation	(63)
Drug resistance	YAP signaling pathway	Promoted EMT, YAP nuclear transportation	(64)
		Merlin/MST/LATS inactivation, ILK/YAP upregulation	(8)
Immune evasion	**\**	PDL1 upregulation	(65, 66)
	**\**	Diminish T cells permeation and migration	(67)

The symbol (\) represents none.

### 3.1 Tumorigenesis of breast cancer promoted by matrix stiffness

Mammary density (MD) is associated with an overall increased lifetime risk of malignancy. Increased mammary density is primarily caused by the deposition of fibrillar collagen (68). It has been shown to result in an increase in stromal stiffness which disrupts the physiologic breast morphogenesis (69, 70). Even a small increase in matrix stiffness results in activation of Rho GTPase and induces collagen matrix contraction to disrupt tissue structure. Rho GTPase also activates the ROCK pathway, which can lead to malignant changes in the breast (49). Collagen cross-linking leads to matrix stiffening promotes integrin aggregation, enhances PI3K activity, and induces oncogene-initiated invasion of epithelial cells (1).

Mammary epithelial cells in cultured soft matrix can grow into normal epithelial tubules; however, in hard matrix, they exhibit an abnormal tumor-like morphology (71). Study of epigenomic changes show that increased stromal stiffness leads to increased nuclear ruffling and lamellipodia-associated chromatin, ultimately inducing a tumor phenotype (72). In a mouse experiment, it was also found that increased matrix stiffness increased mammary tumorigenesis by about three times (73). So, increased matrix stiffness is inextricably linked to breast cancer initiation.

### 3.2 Proliferation of breast cancer cells regulated by matrix stiffness

The uncontrolled proliferation of cancer cells is one of the dominant features of cancer (74). Mesenchymal stem cells (MSC) in a stiff matrix can differentiate into cancer-associated fibroblasts (CAF) with increased expression of the yes-associated protein (YAP) (50). YAP, an important regulatory molecule in the Hippo pathway, is phosphorylated to enter the nucleus to transcribe anti-apoptotic and pro-proliferative genes, thereby regulating cell proliferation and apoptosis and controlling organ size (75–77). In the Wnt pathway, nuclear YAP can promote cell proliferation by up-regulating β-catenin expression (51, 52). In addition, an increase in mammary gland density is often accompanied by an increase in matrix stiffness. Regions with high breast density have increased stromal collagen and epithelial cell contents (78). When NMuMG mammary epithelial cells are cultured on a hard substrate, Wnt3a increases the integrin-linked kinases (ILK)-mediated Frizzled-1 expression and thus promotes epithelial cell proliferation through the integrin signaling pathway (53). Provenzano et al. simulated increased matrix stiffness by increasing the matrix collagen density. They found that matrix stiffness promoted the proliferation of breast cancer cells through FAK-Rho and FAK-Ras-ERK signaling networks (54). Similar conclusions have been reached in animal experiments. Injecting breast cancer cells cultured in a stiffer matrix into mice can form larger tumors (79). In conclusion, matrix stiffness drives breast cancer cell proliferation.

### 3.3 Invasion and metastasis of breast cancer cells regulated by matrix stiffness

**Figure 3:**
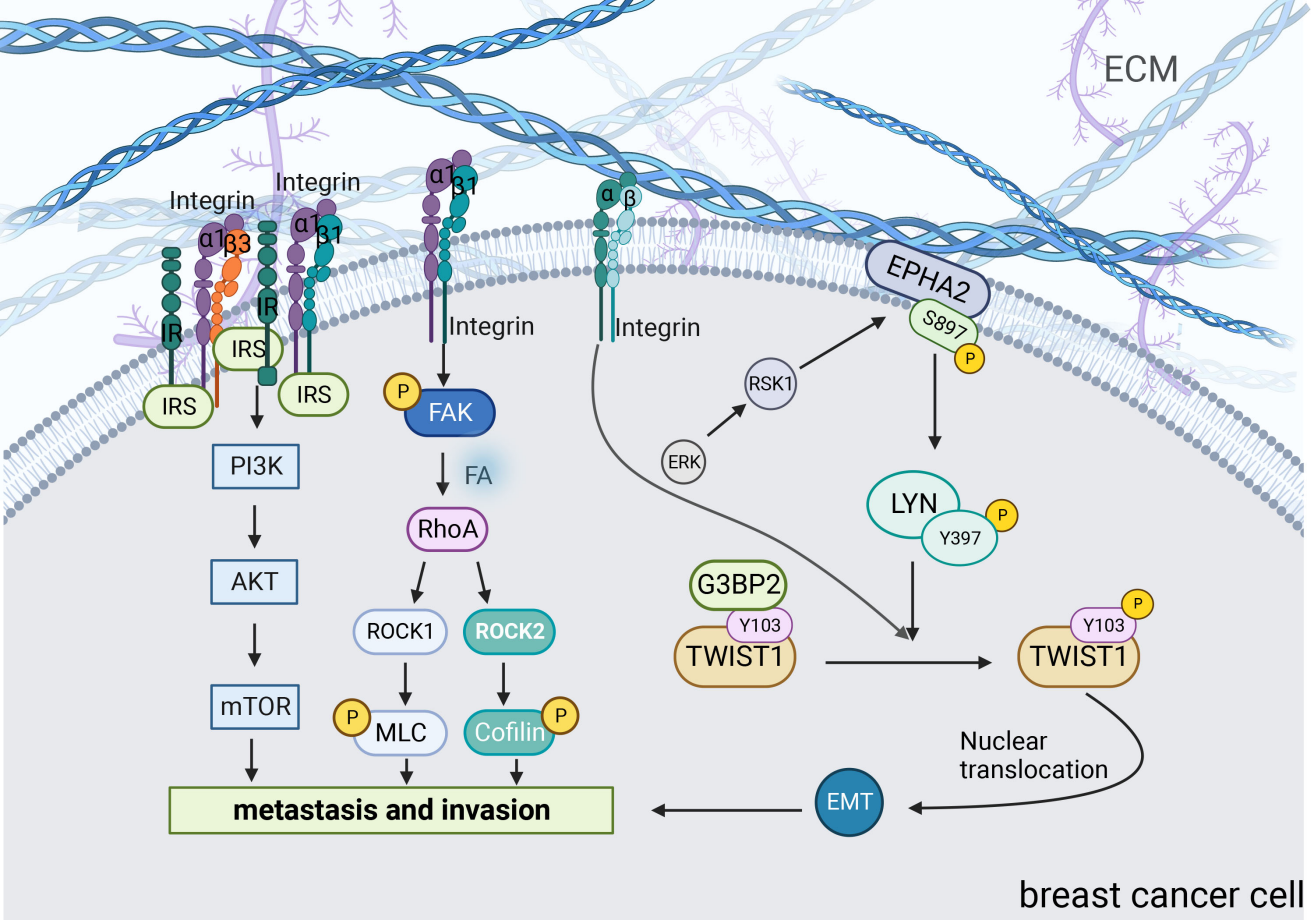
Signaling pathways associated with increased matrix stiffness leading to breast cancer invasion and metastasis. Matrix stiffness activates many mechanoreactive signaling pathways in cells through transmembrane proteins including integrins. Pathways such as PI3K, ROCK and TWIST1 play a major role in this transduction. Central players in these signaling pathways can connect with other molecules and ultimately translate changes in the ECM into relevant biological changes.

Changes in matrix stiffness significantly affect the cytoskeletal structure and ability of breast cancer cells to invade and metastasize. By analyzing PAM50 tumor subtypes, Adam et al. found that compared to the less aggressive luminal A and normal-like subtypes, the more aggressive subtypes such as basal, human epidermal growth factor receptor 2 (HER2), and luminal B, had stiffer matrix and poorer overall survival. They suggested that increased matrix stiffness enhances breast cancer invasion (79). Mechanistically, integrins play important roles in this process (**Figure 3**). Integrin receptors activate insulin receptors (IR) by forming β1 and β3 integrins and IR complexes. IR activates the PI3K/AKT/mTORC1 signaling axis to promote breast cancer cell metastasis (55). Moreover, matrix stiffness can directly activate integrin β1 and focal adhesion kinase (FAK), which accelerates focal adhesion (FA) maturation and induces downstream cascades of intracellular signals in the RhoA/ROCK pathway. ROCK isoforms differentially regulate the RhoA/ROCK1/p-MLC and RhoA/ROCK2/p-cofilin pathways in a coordinated fashion to modulate breast cancer cell motility in a substrate stiffness-dependent manner through integrin β1-activated FAK signaling (58). Moreover, with the activation of EGFR and PLCγ1, the expression of Mena, a protein associated with metastasis in breast cancer, is up-regulated in a stiff matrix. High Mena expression further increases matrix stiffness by depositing fibronectin via α5 integrin (56, 57). In conclusion, integrins are important in promoting the invasive metastasis of breast cancer cells.

One of the key processes that promotes the progression of metastasis in cancer cells is the epithelial-mesenchymal transition (EMT). EMT, the process by which epithelial cells lose polarity, intercellular adhesion, acquire migratory, and invasive properties to become mesenchymal cells, is thought to play a key role in initiating the metastatic cascade response. Thus, EMT allows cancer cells to leave the primary tumor, invade the surrounding ECM, enter the blood and lymphatic vessels, and spread to all body parts (80). When matrix stiffness increasing, cells in the matrix gradually develop an EMT phenotype, indicating that they are more likely to undergo invasive and metastatic spreading (81, 82).

TWIST1 is a basic helix-loop-helix (bHLH) transcription factor that promotes tumor metastasis by initiating EMT and degrading ECT (59, 60). Furthermore, the increase in matrix stiffness causes TWIST1 to move toward the nucleus, directly affecting the EMT program. G3BP2 is a TWIST1 binding protein and tyrosine residue Y103 is present in its binding sequence. In a soft matrix, there is a strong tendency for the two to interact; however, in a stiffened matrix, TWIST1 dissociates from G3BP2 and is transferred to the nucleus (81). Fattet et al. have shown that increased matrix stiffness activates extracellular signal-regulated kinases (ERK) and ribosomal S6 kinase1 (RSK1). Activated ERK/RSK1 phosphorylates the ephrin Receptor EPHA2 at serine 897 (S897). Moreover, phosphorylated EPHA2 activates LYN Kinase to form an EPHA2/LYN complex. This complex phosphorylates Y103 in the TWIST1-G3BP2 binding sequence, leading to TWIST1-G3BP2 dissociation (83). Additionally, it has been demonstrated that during matrix stiffness, activated integrins phosphorylate Y103 through tyrosine kinases, which eventually prevents TWIST1 from binding to G3BP2 (81). Additionally, a study found a positive correlation between TWIST1 expression and tumor stiffness in patients with breast cancer (84). Barriga et al. found that high tissue stiffness promoted EMT triggering neural crest migration, and this study in turn confirmed *in vivo* that higher matrix stiffness increased the propensity of cells to undergo EMT, leading to distant metastasis (85). Generally, increased matrix stiffness promotes breast cancer metastasis by activating the EMT through a mechanical conduction pathway.

In addition, there is a phenomenon in the process of cancer recurrence and metastasis, which is that breast cancer recurrence and metastasis are usually detected in tissues that are softer than normal breast or primary breast tumors (such as bone marrow, liver, brain, and lung) (86). Therefore, the soft microenvironment can promote the survival of disseminated breast cancer cells at the secondary site. When breast cancer cells were cultured on the soft matrix mimicking the site of metastasis, they were found to remain dormant for a long time to escape the killing effects of chemotherapy drugs. Soft matrix can also induce chemical resistance in breast cancer by increasing autophagy, making metastatic breast cancer more difficult to treat (87).

### 3.4 Stemness of breast cancer cells regulated by matrix stiffness

Cancer stem cells (CSC) are a small subpopulation of cancer cells that maintain their self-renewal and undifferentiated abilities. Breast cancer stem cells (BCSC) can self-renew, differentiate, drive tumor progression, and mediate drug resistance and metastasis (88). Pang et al. investigated the relationship between matrix stiffness and BCSC by detecting CSC markers CD44, Nanog, and CD49f. They found that the expression of all these markers increased when the matrix stiffness increased. By comparing the expression of CD44 at different matrix stiffness values, they found that BCSCs were preferentially located in a stiff microenvironment (61).

BCSCs were mainly regulated by ILK. In the presence of increased matrix stiffness, ILK regulates BCSC development via the PI3K/Akt pathway and promotes angiogenesis in tumor cells, ultimately contributing to tumor metastatic spread (61). A recent study found that cells cultured on hard polyacrylamide hydrogels (9 kPa) had a significantly higher proportion of BCSCs compared to cells cultured on soft polyacrylamide hydrogels (0.5 kPa; matching the compliance of normal mammary glands). Exploration of the mechanism revealed that when matrix stiffness was increased, TAZ dissociated from NANOG, promoting the transcription of SOX2 and OCT4, which in turn increased the proportion of BCSCs in the breast cancer and promoted the stemness phenotype of breast cancer (62). In another study, by using three different hydrogels, Matrigel, collagen I, and fibrinogen gels, to simulate three different matrix compositions, collagen, laminin, and fibronectin, respectively, it was found that the increased matrix stiffness due to different matrix compositions had different effects on the stemness of breast cancer (89). Yan Li et al. cultured breast cancer cells using different stiffness of polyacrylamide hydrogels and found that increased matrix stiffness promotes YAP nuclear translocation, which in turn promotes BCSCs maintenance (63). Matrix stiffness may be important for the induction and maintenance of CSC; however, this requires further investigation.

### 3.5 Drug resistance of breast cancer cells regulated by matrix stiffness

Drug resistance is one of the most important factors affecting breast cancer treatment outcomes (90). Improving the sensitivity of breast cancer cells to chemotherapeutic agents is essential to improve the survival rate of patients with breast cancer. In addition, the ability to achieve effective drug concentrations at the tumor site is also essential for the treatment of cancer. Most chemotherapeutic agents are dose-dependent, and chemotherapeutic agents need to pass through the tumor vasculature system, cross the vessel wall to enter, and pass through the interstitial space of the tumor to reach the cancer cells to exert their therapeutic effects. However, when stromal stiffness increases, the extravascular hydrostatic pressure, or interstitial pressure (IFP), increases within the tumor, resulting in inhibited drug extravasation. On the other hand, increased stromal stiffness leads to vascular compression, resulting in inadequate perfusion within the tumor, further reducing drug concentration. More unfortunately, when stromal stiffness is increased, the dense ECM further impedes the effective diffusion of chemotherapeutic agents, ultimately making it difficult to achieve effective concentrations and reducing the efficacy of chemotherapeutic agents (91–93).

The responsiveness of primary breast cancer cells to chemotherapeutic agents is altered after they are removed from the host microenvironment and transferred to hard-surface cultures *in vitro*. The activities of PTX and DOX were strongly correlated with matrix hardness. Substrates that are too hard can reduce the activities of PTX and DOX, leading to drug resistance (94). In addition, the activity of targeted drugs for breast cancer treatment can be influenced by stromal stiffness. Lapatinib is an orally administered small-molecule epidermal growth factor tyrosine kinase inhibitor. It is primarily used to treat HER2(human epidermal growth factor receptor-2)-amplified breast cancer. Furthermore, the ratio of HER2 phosphorylation decrease with increasing matrix stiffness and was negatively correlated with lapatinib insensitivity (95).

Sorafenib is a small-molecule tyrosine kinase inhibitor with anti-angiogenic activity that has been used to treat hepatocellular and renal cancers (96). Hepatocellular cancer cells on stiff substrates show resistance to sorafenib compared to those on soft substrates (97). The same phenomenon has been observed in breast cancer cells (98). Breast cancer cells cultured on harder substrates were more resistant to sorafenib (99).

Moreover, the EMT affects the sensitivity of breast cancer cells to chemotherapy. Notably, increased matrix stiffness promotes the nuclear translocation of YAP, triggering EMT and increasing drug resistance. However, only the MDA-MB-231 cell line showed drug resistance with increased simulated matrix stiffness during the experiment (64). This suggests that the effect of matrix stiffness on drug resistance is related to the cell line. Additionally, matrix stiffness can regulate YAP’s translocation, dephosphorylation, and transcriptional activity by increasing ILK expression, ultimately leading to increased drug resistance in breast cancer cells (8). In summary, targeting matrix stiffness is a prospective strategy for improving the efficacy of chemotherapy.

In addition to chemotherapy, radiotherapy is also an important treatment for breast cancer. One study showed that low doses of radiation had no significant effect on tumor cell migration when matrix stiffness was increased, but when high doses of radiation were changed, tumor cell adhesion increased and migration rate decreased significantly. On soft substrates, low doses of radiation can reduce the migration rate of tumor cells. These results indicate that the radiosensitivity of tumors on hard substrates is dose dependent (100). But the results are not widely accepted. Rieken et al. suggested that radiation promotes tumor migration by inducing integrin overexpression (101). In conclusion, the mechanism of the influence of matrix stiffness on radiosensitivity is still unclear, and some conclusions are still controversial, which may be closely related to radiation dose, radiation time and cell types (93).

In addition, the targeted therapies of breast cancer could also be affected by matrix stiffness. Lapatinib is a targeted drug for the treatment of HER2-amplified breast cancer (102). Increased matrix stiffness leads to YAP overexpression, which in turn modulates the Hippo pathway and reduces the efficacy of lapatinib (95, 103). In conclusion, matrix stiffness has an impact on multiple treatments for breast cancer, including chemotherapy, radiotherapy and targeted therapy.

### 3.6 Immune evasion of breast cancer cells regulated by matrix stiffness

Immunotherapy is a novel modality for the treatment of breast cancer. However, breast cancer is considered a low-immune reactive cancer. The key to immunotherapy is the interaction between the programmed death-1 receptor (PD-1) and programmed death ligand 1 (PD-L1). Previous studies revealed a positive association between high PD-L1 expression and matrix stiffness. High PD-L1 expression in breast cancer is associated with poor prognosis (65, 66). On a physical level, when collagen crosslinks and matrix stiffness increases, T cells have difficulty penetrating the matrix and their ability to migrate in the matrix is greatly diminished, thus limiting the further role of T cells in the tumor (67). Therefore, reversing immune evasion in breast cancer remains a challenge.

## 4 Therapy for breast cancer by targeting matrix stiffness

As the study of matrix stiffness has intensified, new directions for breast cancer treatment have been provided. Matrix targeting in breast cancer can be broadly divided into two types:1) Reducing the source of matrix stiffness. 2) Blocking the effect of matrix stiffness on the downstream pathways (**Table 2**).

**Table 2:** List of conversion therapy drugs.

Categorizations		Drugs	Mechanism	References
Extracellular matrix remodeling enzymes inhibitors	LOX inhibitors	BAPN	Inhibit collagen cross-linking	(104, 105)
		TM	Copper chelator	(25)
	MMPs inhibitors	Prinomastat	MMP -2, -9, -13 and -14 inhibitor	(106)
Targeted drugs	TGF-β	PFD	Inhibition of TGF-βexpression in CAFs	(107)
	Akt	FFE	Reduce phosphorylated Akt	(108)
	HER2-Src-α6β4 integrin	Lapatinib	Inhibit HER2 activity	(109)
Others		MU	Inhibit HA synthesis	(110)

To reduce the source of matrix stiffness and collagen cross-linking, ECM enzymes, such as LOX/LOXLs, MMPs, and CAFs, can be used to directly block the excessive synthesis of certain ECM components. For example, 4-methylumbelliferone (MU) can significantly inhibit the synthesis and accumulation of hyaluronic acid (HA, a matrix component), which promotes tumor cell metastasis (110). β-Aminopropionitrile (BAPN) acts as a LOX inhibitor and suppresses breast cancer proliferation and metastasis by inhibiting collagen cross-linking (25, 104, 105). Tetrathiomolybdate (TM), a LOX inhibitor, belongs to a group of copper chelators that inhibit LOX activity by binding to and depleting copper. A phase IIa TM study is underway in breast cancer patients at an intermediate to high risk of recurrence (25).

Prinomastat is a selective oral matrix MMP -2, -9, -13 and -14 inhibitor. The drug has been shown to prevent angiogenesis and tumor development in a range of preclinical models, including those of colon, breast, lung, melanoma, and glioma (106). Growth factors, including TGF-β, PDGF, and VEGF, can also be used as targets to block the increase in matrix stiffness. Pirfenidone (PFD) is a potent TGF-β inhibitor approved for treating pulmonary and renal fibrosis (111). For example, Hamidreza et al. showed that PFD reduced breast cancer epithelial-mesenchymal transition and globule formation by targeting CAFs (107).

Downstream receptors of matrix stiffness, such as integrins, FAK, Rho GTPase, and AKT, can be used as therapeutic targets. Seon-Ok Lee et al. found that fomes fomentarius ethanol (FFE) could inhibit MDA-MB-231cells motility and growth, by reducing the expression of MMP-9 and phosphorylated Akt (108). Furthermore, the complex formation of HER2-Src-α6β4 integrin influences the targeted therapy with lapatinib. Cuiying Liu et al. explored how stiffness regulated the response of breast cancer cells to lapatinib. They found that, on the stiff substrate, the HER2 is difficult to combine with β4 integrin molecules, constructing fewer complexes of HER2-Src-α6β4 integrin. Consequently, free HER2 molecules were inhibited by lapatinib. In addition, as early as 2002, the concept of “biomechanopharmacology” was first proposed (109). The development of this field will provide new ideas for future treatments.

## 5 Conclusions and perspectives

The role of the ECM in tumorigenesis has been increasingly studied, and changes in matrix stiffness have also been considered as factors contributing to disease development. This review begins with an introduction to the mechanical microenvironment in breast cancer. We then elaborated on the effect of extracellular matrix stiffness on breast cancer’s biological behavior and signaling pathway. Finally, we discuss the transformation treatments for matrix stiffness in breast cancer.

In addition, several questions remain unanswered. Can matrix hardness be integrated into clinical research? Is there an interaction between the various mechanical stimuli? Can mechanical stimuli such as matrix stiffness be measured quantitatively? Are there signaling pathways other than those mentioned above? Research into the effects of matrix hardness on signaling pathways is only beginning, and the effects of matrix stiffness on biological pathways, such as transcription, post-transcriptional modification, translation, and post-translational modification, need to be further investigated. In addition, we also noted that antibody-drug conjugates (ADCs) are gradually becoming a novel treatment for breast cancer. However, studies on the aspect of ADCs related to matrix stiffness are still relatively scarce, and further studies are needed to explore the relationship between the two subsequently. Although studies on the effects of matrix stiffness on breast cancer are already underway, our understanding of the mechanisms involved is limited to the tip of the iceberg. We will be able to develop new therapeutic options through a better understanding of matrix stiffness. We believe that concerted efforts by researchers are required to address these questions.

## Author contributions

RX: Writing – original draft. JW: Writing – review & editing. QD: Writing – review & editing. PY: Writing – original draft.
